# Bio-priming with salt tolerant endophytes improved crop tolerance to salt stress *via* modulating photosystem II and antioxidant activities in a sub-optimal environment

**DOI:** 10.3389/fpls.2023.1082480

**Published:** 2023-03-09

**Authors:** Khadija Irshad, Zamin Shaheed Siddiqui, Jianjun Chen, Yamna Rao, Hafiza Hamna Ansari, Danish Wajid, Komal Nida, Xiangying Wei

**Affiliations:** ^1^ Department of Botany, Stress Physiology Phenomic Centre, University of Karachi, Karachi, Pakistan; ^2^ Mid-Florida Research and Education Center, Environmental Horticulture Department, Institute of Food and Agricultural Science, University of Florida, Apopka, FL, United States; ^3^ Institute of Oceanography, College of Geography and Oceanography, Minjiang University, Fuzhou, China

**Keywords:** Chlorophyll ‘a’ fluorescence, bacterial priming, wheat, mung bean, ionic stress, response

## Abstract

Abiotic stress is one of the major constraints which restrain plant growth and productivity by disrupting physiological processes and stifling defense mechanisms. Hence, the present work aimed to evaluate the sustainability of bio-priming salt tolerant endophytes for improving plant salt tolerance. *Paecilomyces lilacinus KUCC-244* and *Trichoderma hamatum Th*-*16* were obtained and cultured on PDA medium containing different concentrations of NaCl. The highest salt (500 mM) tolerant fungal colonies were selected and purified. *Paecilomyces* at 61.3 × 10^-6^ conidia/ml and *Trichoderma* at about 64.9 × 10^-3^ conidia/ml of colony forming unit (CFU) were used for priming wheat and mung bean seeds. Twenty- days-old primed and unprimed seedlings of wheat and mung bean were subjected to NaCl treatments at 100 and 200 mM. Results indicate that both endophytes sustain salt resistance in crops, however *T. hamatum* significantly increased the growth (141 to 209%) and chlorophyll content (81 to 189%), over unprimed control under extreme salinity. Moreover, the reduced levels (22 to 58%) of oxidative stress markers (H_2_O_2_ and MDA) corresponded with the increased antioxidant enzymes like superoxide dismutase (SOD) and catalase (CAT) activities (141 and 110%). Photochemical attributes like quantum yield (F_V_/F_M_) (14 to 32%) and performance index (PI) (73 to 94%) were also enhanced in bio-primed plants in comparison to control under stress. In addition, the energy loss (DI_O_/RC) was considerably less (31 to 46%), corresponding with lower damage at PS II level in primed plants. Also, the increase in I and P steps of OJIP curve in *T. hamatum* and *P. lilacinus* primed plants showed the availability of more active reaction centers (RC) at PS II under salt stress in comparison to unprimed control plants. Infrared thermographic images also showed that bio-primed plants were resistant to salt stress. Hence, it is concluded that the use of bio-priming with salt tolerant endophytes specifically *T. hamatum* can be an effective approach to mitigate the salt stress cosnequences and develop a potential salt resistance in crop plants.

## Introduction

The twin goals of ensuring global agricultural productivity and its execution in a sustainable manner are challenged due to the increased incidence of ecological catastrophes (Ebert and Engels, 2020). As a result, our agriculture system is frequently subjected to both biotic and abiotic stress. In the last few decades, a number of studies have been reported the effect of abiotic and biotic stressors on crops (Chinnusamy et al., 2005; Kwon et al., 2009; Fizza et al., 2021; Ansari et al., 2022), highlighting the alternate means of controlling the negative impacts of such stressors and sustain plant growth in a sub-optimal environment. Moreover, out of many environmental fluctuations, soil salinization has become a fundamental enigma as it has been encountered in all climates. The assault of this salinity stress, which is mainly caused by sodium ions, can be observed in the germination, growth, development, and reproduction of the crop (Mahmood et al., 2021). Hence, soils are rendered hypersaline due to the prevalence of NaCl by natural or anthropogenic means, which decreases crop production by more than 20% (Porcel et al., 2012). In response to salt stress, plants show plasticity in terms of periodic adjustment like osmolyte synthesis due to physiological modifications in their defensive metabolism (Nephali et al., 2021). However, the strategies to adapt salt tolerance in crops have become insufficient to overcome extreme salinity (Augé et al., 2014). Thus, to mitigate the salt stress and sustain the modern agriculture system, various biotechnological approaches have been employed to ensure crop productivity.

Among such approaches, bio-priming has been considered an innovative and sustainable method for alleviating plant salt stress. Seed bio-priming is a strategy of seed treatment (seed priming) for regulating plant growth, managing stress, and improving seed germination (Sarkar et al., 2021). Moreover, seed priming alone (osmo-priming, matrix priming) or in combination with a low dosage of biocontrol agents have been reported to increase the germination rate, uniformity and sustainability of plant growth and development under sub-optimal environment (Johnson and Puthur, 2021). However, Seed priming *via* conventional and specifically chemical means impaired the soil ecosystem, which creates fluctuations in the food chain. Therefore, seed bio-priming with plant growth-promoting microbes (PGPM) that are naturally colonized around the root zone of the plants has a great potential to increase the plant’s performance in a suboptimal environment (Dimkpa et al., 2009).

In addition, it is currently being recognized that the application of endophytes offers a great potential to reduce the abiotic and biotic stress in plants. Lately, the application of endophytes to reduce the hypersaline stress in plants has also been reported (Sandhya et al., 2009; Yao et al., 2010; Verma et al., 2021). Several studies suggested that the endophytes sustained growth by increasing the uptake of nutrients such as zinc, phosphorus, boron and copper and making other nutrients available to plants in a saline-sodic soil (Sarma et al., 2015; Liu et al., 2017).

*Paecilomyces lilacinus *and *Trichoderma hamatum* are endophytic saprophyte fungus that can be found in different soil types and have the ability to grow in a broad range of soil pH having sodium ions. *P. lilacinus* is effectively used to control nematode growth as it has the ability to penetrate and destroy the embryo. Similarly, *T. hamatum* is a beneficial endophytic plant symbiont, compared to *P. lilacinus* which is widely used to control fungal diseases in crop plants (Afzal et al., 2013). Some reports indicate that that *Trichoderma* enhanced the tolerance to abiotic stress in plants (Shoresh et al., 2010; Estrada et al., 2013). However, the role of *P. lilacinus* in plants to enhance stress tolerance against abiotic stress has not been reported so far. Hence, the present study aimed to probe the application of *P. lilacinus* and *T. hamatum* as an effective bio-priming agent in crop plants against hypersaline environment.

Plant photosynthesis coupled with defense mechanisms are the prime physiological modulations that indicate the health status of the crops. The thorough analysis of the photosynthetic apparatus *via* non-destructive approach like chlorophyll fluorescence can mimic the real time changes in perturbation and light harvesting efficiency of the photosynthetic membrane. Furthermore, light harvesting complexes and reaction centers of PS II are not only true source of energy production but also plays a crucial role to stress tolerance under abiotic stresses. Therefore, the present study evaluated the sustainable role of isolated endophytes through seed-priming on photo-physiology, light harvesting efficiency, energy fluxes, and subsequent antioxidant system in two important crops, under a suboptimal environment. Also knowing that the energy exploitation in the photosynthetic apparatus of bio-primed plants during salt stress tolerance has not been documented so far. Likewise the application of *T. hamatum* and *P. lilacinus* as a bio-priming agent to enhance salt tolerance in plants is yet to be studied In essence, the current research was designed to scrutinize the energy distribution inside the photosynthetic membrane by non-destructive means to explicate the energy source for the induction of salt tolerance in plants due to bio-stimulating natural colonizers i.e., *T. hamatum* and *P. lilacinus*.

## Materials and methods

### Seed source and selection

Seeds of Wheat *(Triticum aestivum)* and Mung bean *(Vigna radiata)* were collected from the Stress Physiology Phenomic Centre, Department of Botany, University of Karachi, and surface sterilized into 10% NaClO (sodium hypochlorite) for 3 min to remove the surface fungus and dust. Seeds were then thoroughly washed with distilled water to remove NaClO traces.

### Collection and purification of beneficial endophytic fungi

**Figure 1:**
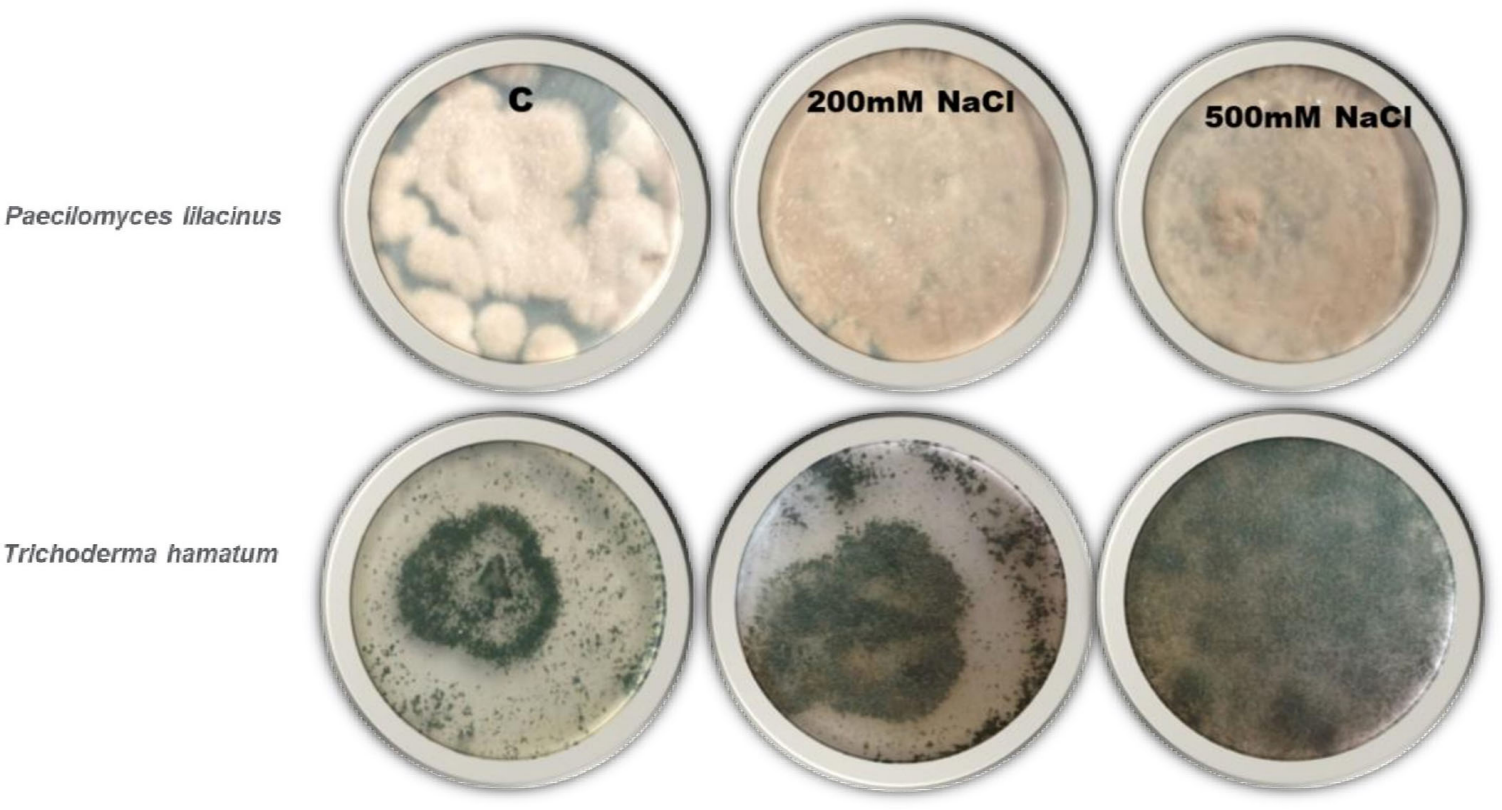
Endophytes culture on high saline medium to use in plant. Highest salt tolerance endophytes culture was used in further study.


The plant-beneficial fungal endophytic fungi *P. lilacinus* and *T. hamatum* were obtained from Karachi University Culture Collection (KUCC) and purified on PDA (Potato Dextrose Agar) with several replicates. Saline medium of PDA was prepared to examine the salt tolerance of *P. lilacinus* and *T. hamatum*, having several concentrations of NaCl (100, 200, 300, 400, and 500 mM) in its composition. These sets were kept at room temperature 30-34 ± 2°C for 7 days to select salt-tolerant endophytic strains and later it was used for further study (**Figure 1**). The Colony-forming unit (CFU) was maintained at 61.3 × 10^-6^ Conidia/ml of *Paecilomyces* and about 64.9 × 10^-3^ Conidia/ml of *Trichoderma c*olony forming units (CFU) per milliliter for liquid as:

$$Cfu/ml=\frac{No.\;of\;colonies\;\times \;dilution\;factor}{The\;volume\;of\;the\;culture\;plate}$$

### Inoculation of fungal endophytes by seed priming technique

The endophytic fungi *P. lilacinus* and *T. hamatum* were inoculated in plants by seed bio-priming technique as described by Saeid et al. (2018). Seeds of Wheat and Mung bean were selected for the inoculation of endophytes. The fungal suspension was prepared from pure PDA cultures by adding 10 ml of sterile distilled water into fungal plates. Plates were slightly scratched by a wire loop and fungal suspension was poured into a beaker (the process was repeated twice). The final volume was made up to 100 ml with sterile distilled water to make the stock. From the fungal spore stock, 25 ml was taken and made up the volume up to 100 ml with sterile distilled water to prepare 25% fungal suspension. Later, the surface sterilized and dried seeds of both crops were treated by soaking in the spore suspensions prepared for different time intervals (5, 10, and 15 min). The seeds were dried under a sterile air stream in laminar air flow for 2 h (Singh et al., 2013).

### Experimental design and stress application

The experiment was conducted at the Stress Physiology Phenomic Center, Department of Botany, University of Karachi, Pakistan. Under natural environmental conditions, the experiments were organized in a completely randomized design to analyze endophytic symbiosis with the crop plant. Two sets of experiments were conducted, 1) Seeds without inoculation of *P. lilacinus* and *T. hamatum* and 2) Seeds with inoculation of *P. lilacinus* and *T. hamatum*. Ten treated seeds were sown per pot having 1 Kg of soil and allowed to germinate. The composition of the soil is 80.5% sand particles, 7.1% silt and 8.1% clay, 4.10% organic carbon, 0.83% total nitrogen, pH 7.6, and electrical conductivity was 1.7 dS.m^-1^. Wheat and Mung bean were allowed to grow at an average day-night temperature of 33 ± 4°C to 22 ± 3°C. Twenty days old inoculated and un-inoculated seedlings were treated with different salt concentrations by gradual increment method to reach 100 and 200 mM NaCl. In this regard, 50 mM for 200 mM and 25 mM for 100 mM was given on alternate days. The moisture level was maintained by adding up water as stated by Umar and Siddiqui (2018). The whole setup of the experiment was repeated with four replicates of treatments and controls. The plants were exposed to saline treatments for 7 days and later plants were harvested.

### Relative water content

For the calculation of Relative water content (RWC) Barrs and Weatherley (1962) method was applied with some modifications. Randomly selected leaves of each control and treated samples of an area of 4 × 2 cm^2^ of wheat and 1.2 cm^2^ of Mung bean were excised from the mid-veins and the edge section and fresh weight (FW) was recorded. Later, leaves were kept in Petri plates of 90 mm diameter for12 h, which contain distilled water. Afterward, the leaves samples were taken off the Petri plate and turgid weight (TW) were recorded. For the measurement of the dry weight (DW), leaves samples were oven dried at 80°C for 48 hours. RWC was calculated by using the formula:


$$RWC=\frac{FW-DW}{TW-DW}\;\times 100$$

### Stomatal conductance and chlorophyll content index

For the observation of stomatal conductance, young randomly selected leaves of Wheat and Mung bean from each treated and control intact plant was used between 9:00 A.M. and 11:00 A.M. For this investigation, Decagon Leaf Porometer (Model SC-1) was used, and data were recorded from the middle and lower part of the leaf surface. The stomatal conductance of leaf was expressed as mmole m^-2^s^-1^. Similarly, the chlorophyll content index (CCI) of young leaves of each treated and control intact plant leaf was recorded between 9:00 A.M. and 11:00 A.M. Chlorophyll Content Meter CCM-200; Opti-Sciences Inc., Hudson, NH, USA was used. The average values of ten leaves of each replicate were used to show in bar graphs.

### Photochemical traits of photosystem II

For the photochemical traits of Photosystem II assessment, chlorophyll fluorescence was recorded by using as Opti-Sciences Fluorometer (Model OS-30 p^+^; Hudson, USA). For the analysis, the youngest and fully expended leaves were selected between 9:00 A.M. and 11:00 A.M. From intact plants, leaves were clipped for 60 min for dark-adapted measurement from each treatment and control plant. Light-adapted quantum yield was recorded under a normal day-light environment. Performance index (PI_ABS_), Original (F_O_), and maximum (F_M_), the dark-adapted quantum yield of PS II photochemistry was calculated by the ratio of variable to maximum fluorescence (F_V_/F_M_), photochemical quenching (qP), and JIP test data was used to calculate as described by Strasser et al., 2010; Stirbet and Govindjee, 2011 (**Supplementary Table 1**).

### IR thermal images

FLIR-E5 (FLIR Systems, USA) was used before harvesting. IR thermal sensor observed the infra-red thermography from each Wheat and Mung bean treated and control plant. Before the measurement, the system was optimized for 60-90 min, and later on, images were taken. A computerized report was generated using FLIR Software 2.10 after transferring the images into the computer.

### H_2_O_2_ content

Total hydrogen peroxide (H_2_O_2_) content was estimated according to the method described by Velikova et al. (2000). Freshly harvested leaf samples were homogenized in 3 ml of 0.1% (w/v) trichloroacetic acid (TCA) in an ice bath. Afterward, homogenate was centrifuged at 12000 rpm for 15 min. Later on, 0.5 mL of 10 mM phosphate buffer (pH 7.0) and 1 ml of 1 M potassium iodide (KI) were mixed with 0.5 ml of supernatant. Optical density of the supernatant was taken at 390 nm. H_2_O_2_ content was estimated with reference to a standard curve and expressed in mmole g^-1^ FW.


$$H_{2}O_{2}\;Content=Ve\;\times R\;\times \frac{D.F}{Vs}\;\times \;W\;$$

Where,

Ve _=_ Volume used for the estimation, R _=_ Reading from the standard curve, D.F _=_ Dilution factor, Vs _=_ Volume of extract, W _=_Weight of leaf sample.

### Malondialdehyde content

Lipid peroxidation in the leaf tissues was observed by Dhindsa et al. (1981), the amount of malondialdehyde (MDA) produced by the reaction of Thio-barbituric acid (TBA). Freshly harvested leaves samples of 0.25 g were homogenized with 0.1% trichloroacetic acid (TCA) in a pestle and mortar and centrifuged at 10,000 rpm for 5 min. 1mL supernatant was added into 4 ml of 20% TCA containing 0.5% TBA. The mixture was heated for 30 min in a water bath at 95 °C and allowed to cool. Absorbance was recorded at 532 and 600 nm. MDA-TBA extinction co-efficient was recorded at 532 nm.


$$Con\mathrm{c.}\;\;of\;MDA\;(µM)=\frac{\left(A_{532}-A_{600}\right)}{155}$$

### Antioxidant enzymes activity

Leaf sample of 500 mg in liquid nitrogen (5°C) was homogenized with 10 ml of abstraction buffer (Tris-HCl pH 6.8, 10 ml DDT, 0.1 mM EDTA, 50 mg PVP) for enzymatic antioxidant evaluation. The mixture was centrifuged at 15,000 rpm for 10 mins to estimate total protein by the method described by Bradford (1976). The antioxidant enzymes i.e., Superoxide Dismutase (EC # 1.15.1.1) and catalase (EC # 1.11.1.6) was measured by the method of Beyer and Fridovich (1987) and Patterson et al. (1984), respectively.

### Statistical analysis

The data generated from the treated and control groups were subjected to statistical analyses using the software SPSS Version 20 (IBM, United States). The Bonferroni *Post- hoc* test was applied to differentiate significant differences among the mean values of different treatments and presented as small alphabets on the bar graphs (*p< 0.05*).

## Results

### Morphological response of plants against different priming treatments

**Figure 2:**
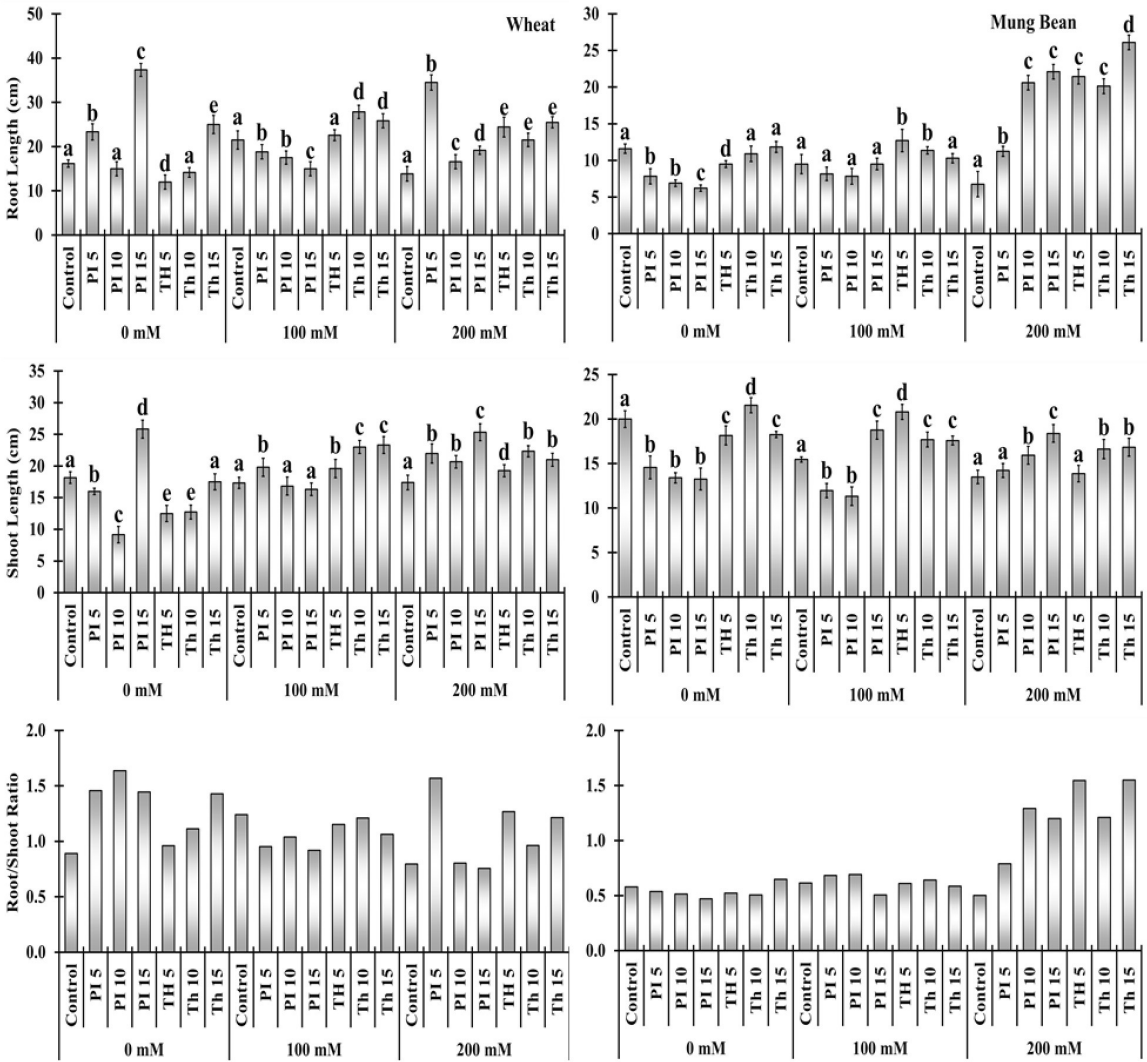
Effects of bio- priming with fungal endophytes on Root Length (RL), Shoot Length (SL) and Root/Shoot ratio of wheat and mung bean grown under saline environment *Note*: The symbols on the horizontal axis represents: Control: Seeds without priming, Pl _=_ Seed priming with *Paecilomyces lilacinus*, *Th*_=_ Seed priming with *Trichoderma hamatum* 5, 10, 15 = duration of bio-priming in minute, 0, 100 & 200 mM NaCl concentration. On bars, vertical lines represent ± Mean Standard Error (S.E) and similar alphabets represents non-significant difference between the the means of treatment at *p<0.05*.

In the sub-optimal environment, seedling length of wheat and mung bean plants was significantly reduced compared to control (**Figure 2**). It was evident from the data that the maximum reduction in root and shoot length was observed in wheat (13.83 and 17.4 cm) and mung bean (6.77 and 13.5 cm) plants when exposed to 200 mM salt stress. However, bio-priming with *T. hamatum* and *P. lilacinus* alleviates the salt stress and thus increases the seedling length of wheat from 26 to 149% and mung bean from 5 to 216% (**Supplementary Table 2**). It was observed that bio-priming agents results in a more profound increase in the root length as compared to the shoot length. However, general trend shows that the increase in priming duration such as 5, 10, and 15 minutes had a positive impact on the shoot length in both plants compared to root length. Unlike, the percentage of root length with respect to time duration was slightly increased in bio-primed treated wheat (20 to149%) and substantially increased in mung bean plants (66 to 285%) under 200 mM salt stress (**Figure 2**, **Supplementary Table 2**). Among all the treatments, the highest root-to-shoot ratio was observed in mung bean plants when it was primed with *T. hamatum* (141 to 209%) salt stress, followed by *P. lilacinus* (57 to 157%) under 200 mM salt stress. However, the root to shoot ratio was comparatively much lower in wheat plants compared to mung bean (**Figure 2**).

### Chlorophyll content index and stomatal conductance

**Figure 3:**
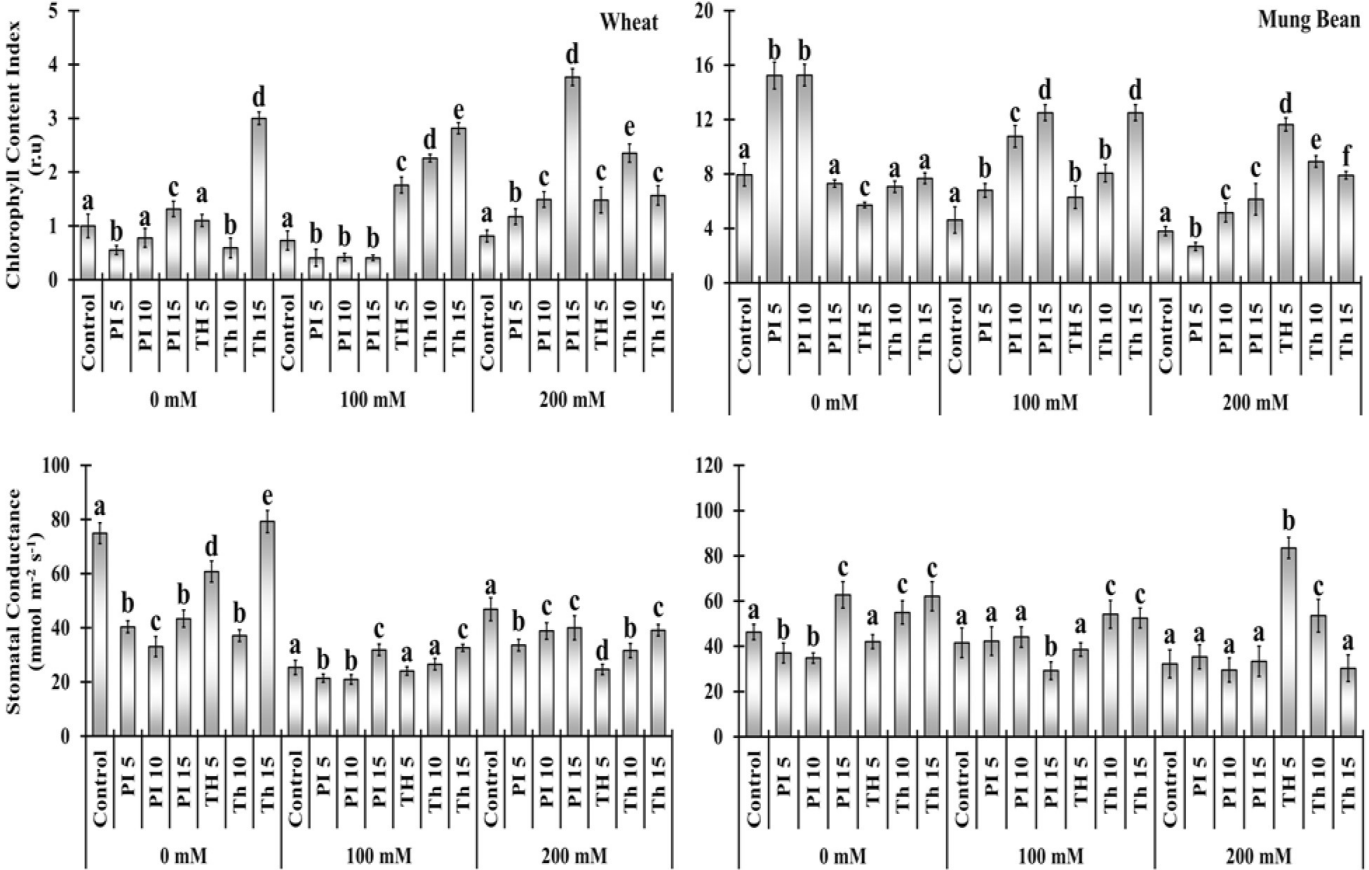
Effects of bio-priming on chlorophyll content index (CCI) and stomatal conductance (gs) of wheat and mung bean plants grown under saline environment. The symbols on the horizontal axis represents: Control: Seeds without priming, Pl _=_ Seed priming with *Paecilomyces lilacinus*, *Th*_=_ Seed priming with *Trichoderma hamatum* 5, 10, 15 = duration of bio-priming in minute, 0, 100 &200 mM NaCl concentration. On bars, vertical lines represent ± Mean Standard Error (S.E) and similar alphabets represents non-significant difference between the means of treatment at *p<0.05*.

Salt stress substantially reduced the chlorophyll content index (CCI) and stomatal conductance of the unprimed plants in comparison to the primed. Bio-priming with *T. hamatum* significantly increased CCI over control in wheat plants with an increase in priming duration, which was about 141 to 285% under 100 mM and 81 to 189% in 200 mM salinity (**Figure 3**, **Supplementary Table 2**). Moreover, *P. lilacinus* priming had a substantially negative effect on wheat plants at 100 mM salt stress, displaying a decline in CCI percentage over control (-43, -42 and -44%) but substantially increased the CCI content of wheat plants over control under 200 mM salt stress (44, 83, and 362%). *P. lilacinus* expressed more profound effect on the mung bean plants compared to wheat, had significantly increased the CCI at both 100 and 200 mM salt stress (47 - 170%and 35 - 61%).

Two types of the consequential stimulated regime by priming agents in wheat and mung bean plants regarding stomatal conductance were observed under extreme salinity (200 mM). Stomatal conductance was significantly decreased in wheat plants over the control when primed with *T. hamatum*, (-47, -32, and -16%) and with *P. lilacinus* (-28, -17, and -14%). In contrast, in mung bean plants, both priming agents substantially increased the stomatal conductance over the control (9, -8, 3, 159, 65 and -6%) with some exceptions under 200 mM salt stress respectively (**Figure 3**, **Supplementary Table 2**).

### Oxidative damage markers

**Figure 4:**
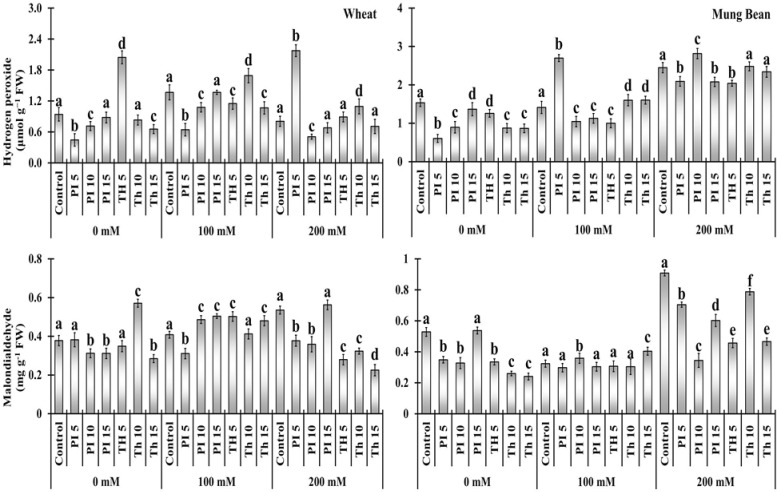
Effects of bio-priming with fungal endophytes on hydrogen peroxide (H_2_O_2_) and Malondialdehyde (MDA) contents of wheat and mung bean grown under saline environment. The symbols on the horizontal axis represents: Control: Seeds without priming, Pl _=_ Seed priming with *Paecilomyces lilacinus*, *Th*_=_ Seed priming with *Trichoderma hamatum* 5, 10, 15 = duration of bio-priming in minute, 0, 100 & 200 mM NaCl concentration. On bars, vertical lines represent ± Mean Standard Error (S.E) and similar alphabets represents non-significant difference between the means of treatment at *p<0.05*.

Elevated level of H_2_O_2_ and MDA in un-primed plants indicates that salt stress relatively increased the oxidative stress. Bio-priming alleviates the stress in wheat and mung bean plants as the oxidative damage was relatively lower than in control plants. Under 100 mM salt stress, H_2_O_2_ was relatively lower in wheat plants primed with *T. hamatum* (-15, 23, and -22%) and *P. lilacinus* (-52, -21, and -12%) with some exceptions. (**Figure 4**, **Supplementary Table 2**). Moreover, the MDA content among the primed plants was considerably lower in both wheat and mung bean plants in comparison to the control plants. It was evident from the data that MDA content was considerably decreased with the priming of *T. hamatum* (-47, -39, and 58%) than with *P. lilacinus* (-29, -32, and 4.98%) in wheat plants under high salinity (200 mM).

### Photochemical attributes

**Figure 5:**
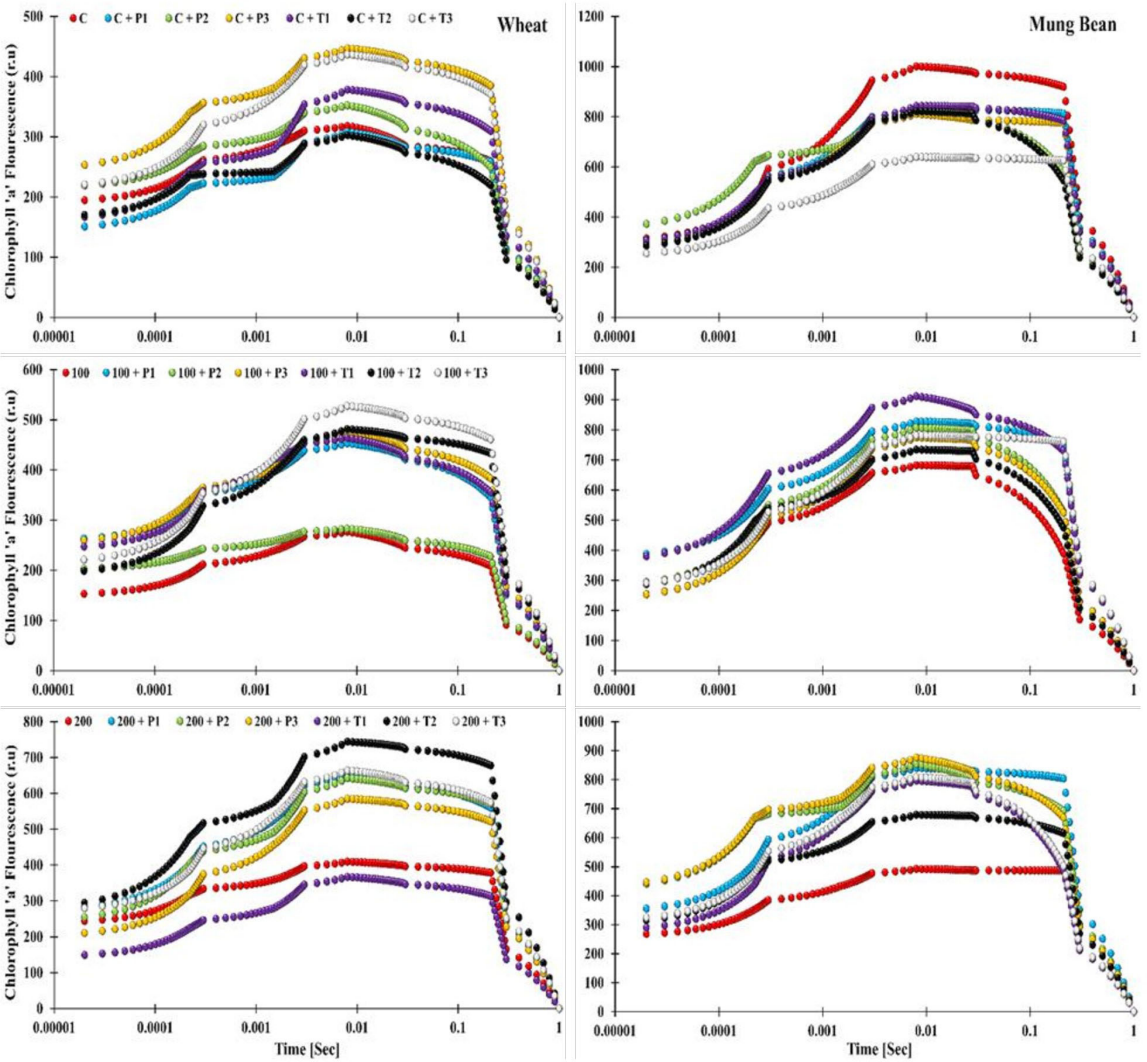
Effects of bio-priming with fungal endophytes on OJIP transient curve of wheat and mung bean grown under saline environment. The symbols on the horizontal axis represents: Control: Seeds without priming, Pl = Seed priming with *Paecilomyces lilacinus*, Th= Seed priming with *Trichoderma hamatum* 5, 10, 15 = duration of bio-priming in minute, 0, 100 & 200 mM NaCl concentration.

Salt stress results in a significant decrease in the performance index (PI) and an increase in the dissipation per reaction center (DI_O_/RC) in wheat and mung bean plants, which was later overcome by bio-priming. Results showed that under 200 mM salt stress, the highest PI was observed in mung bean plants primed with *T. hamatum* (94%) followed by *P. lilacinus* (73%) over the control (unprimed plants). Likewise, a similar trend was observed regarding the maximum quantum yield of PS II (F_V_/F_M_) in mung bean plants (32 and 26%) in comparison to the un-primed stress plants. In wheat plants, priming of *P. lilacinus* caused the highest PI and F_V_/F_M_ (455 and 18%), followed by *T. hamatum* (357 and 14%) under 200 mM salt stress. However, one way to assess the plant’s performance is to observe the release of absorbed energy, which indicates the performance of the plant under stress conditions. In the present study, we found that dissipation per reaction center (DI_O_/RC) was significantly decreased due to the priming in both wheat (-31, -42, and -35%) and mung bean (-39, -42, and -46%) under the extreme salinity level (200 mM) (**Figure 5**, **Supplementary Table 2**).

**Figure 6:**
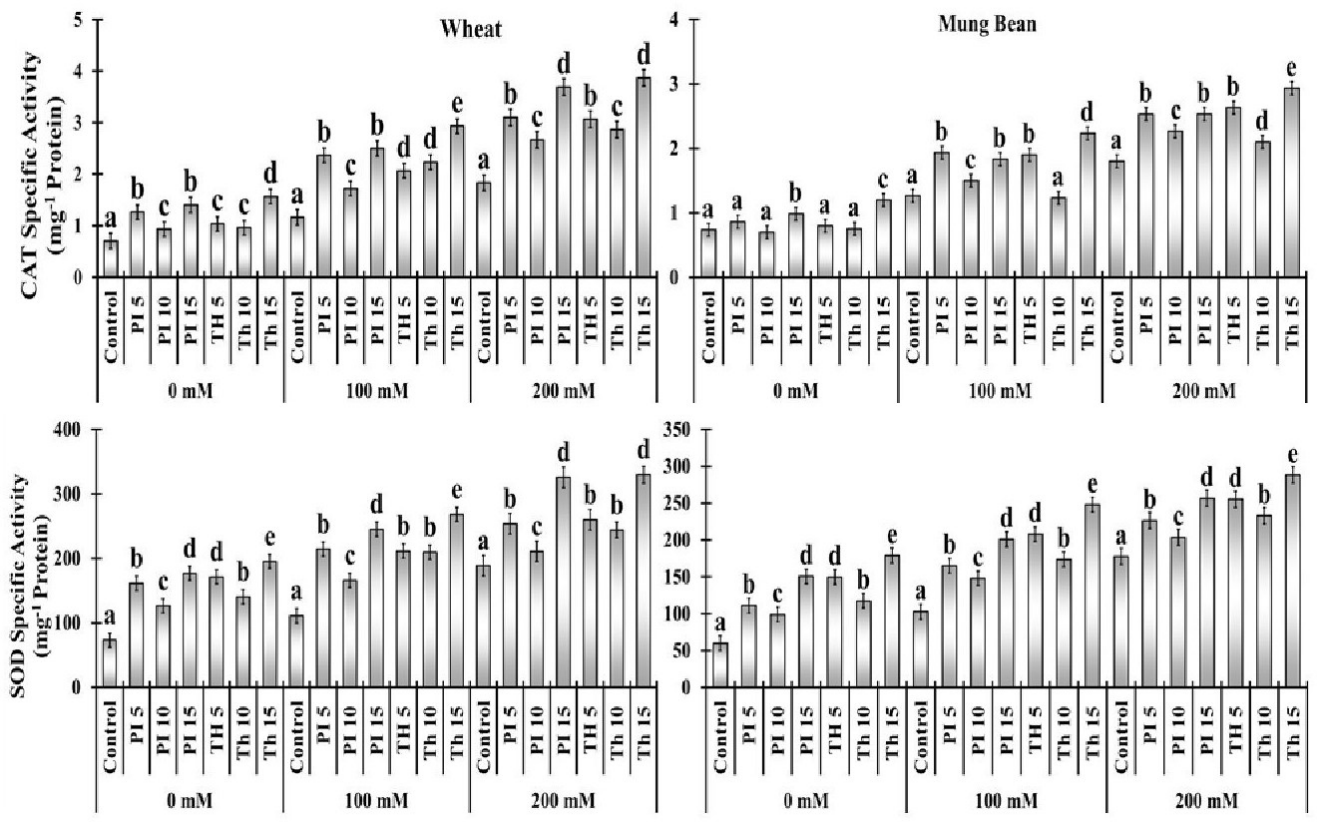
Effects of bio-priming with fungal endophytes on Catalase Specific Activity (CAT) and Superoxide Dismutase Specific Activity (SOD) contents of wheat and mung bean grown under saline environment. The symbols on the horizontal axis represents: Control: Seeds without priming, Pl = Seed priming with *Paecilomyces lilacinus*, Th= Seed priming with *Trichoderma hamatum* 5, 10, 15 = duration of bio-priming in minute, 0, 100 & 200 mM NaCl concentration. On bars, vertical lines represent ± Mean Standard Error (S.E) and similar alphabets represents non-significant difference between the means of treatment at *p<0.05*.

The OJIP induction curve analysis showed the effect of salt stress as the increase in salinity level (from 0, 100, and 200 mM) caused the decline in the fluorescence intensity (OJIP curve) of the un-primed wheat plants. Highest peaks of the induction transients were observed among the bio-primed plants under both non-stress and stress conditions (*T. hamatum* and *P. lilacinus)*, while the lowest curve was displayed by the unprimed 200 mM stress plants. However, one striking pattern was observed among the OJIP curve of plants primed with *T. hamatum* (10 min priming duration) in wheat and mung bean plants. In wheat plants under control (unstressed) conditions, the aforementioned treated plants showed the lowest induction curve, which was moderately increased under 100 mM salt stress and led to the highest peak of all under 200 mM salt stress. In contrast, a complete revert pattern was observed in mung bean plants. *T. hamatum* (10 mins) primed plants had the highest induction curve values in the control environment, which then decreased to moderate values and then further decreased to a lower curve in the high salinity (200 mM) environment (**Figure 6**). Moreover, in mung bean plants, the lowest curves were still attributed to the un-primed plants, showing the stress retardation in the photosynthetic machinery of the mung bean plants. The highest curves were exhibited by the plants primed with *P. lilacinus* under 200 mM stress.

### Antioxidant enzymes

**Figure 7:**
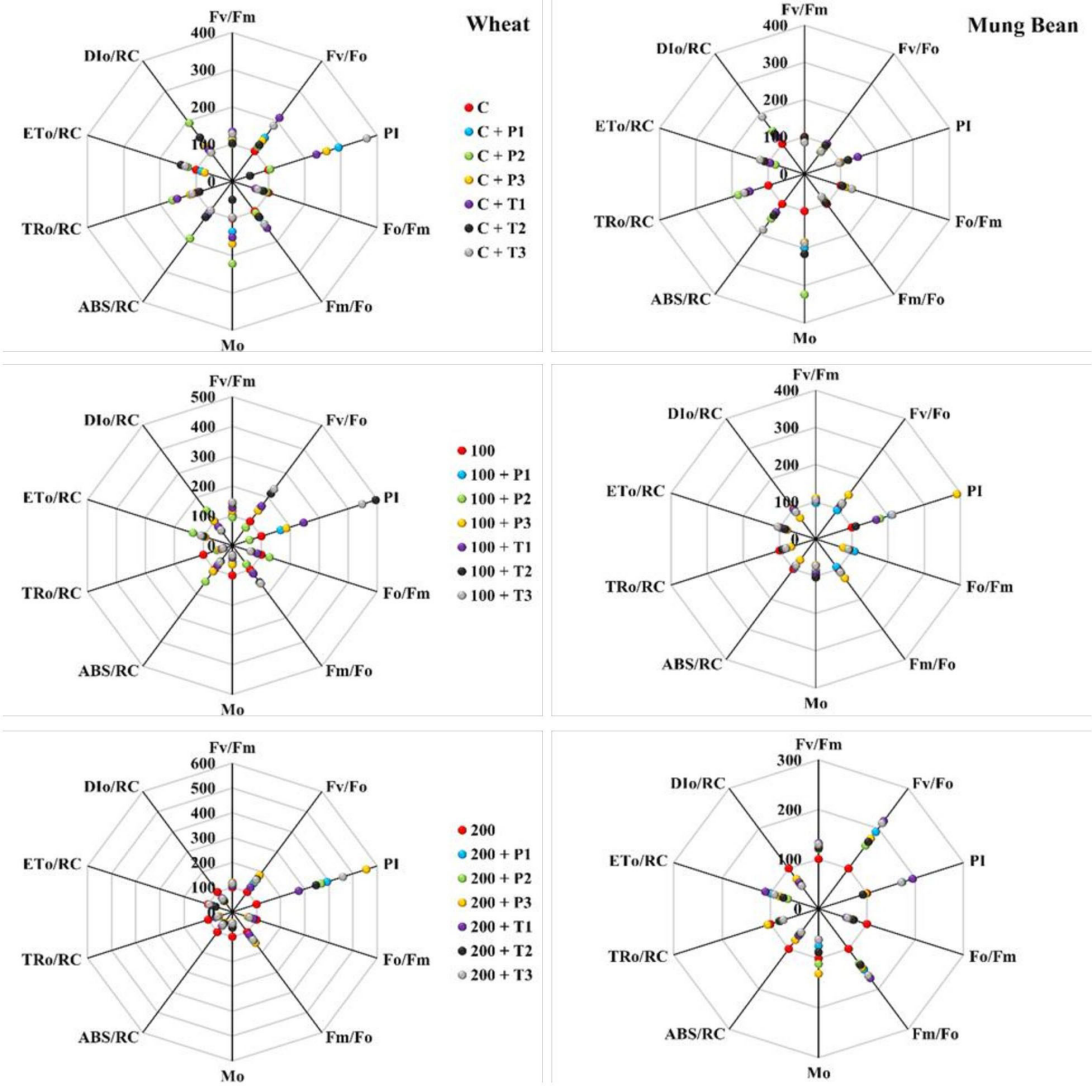
Effects of bio-priming on maximum quantum yield of PSII (*FV/FM*), activity of water splitting complex on donor site of PSII (*FV/F_O_*), performance index (PI), quantum yield of energy dissipation (*F_O_/FM*), electron transport rate through PSII (*FM/F_O_*), approximated initial slope of fluorescence transient (*M_O_*), absorption per reaction centre (*ABS/RC*), trapping per reaction centre (*TR_O_/RC*), electron transport per reaction centre (*ET_O_/RC*) and dissipation per reaction centre (*DI_O_/RC*) of wheat and Mung bean grown under saline environment. The values of the parameters are expressed as percentage increase or decrease over the control (considered as 100). The symbols on the horizontal axis represents: Control: Seeds without priming, Pl = Seed priming with Paecilomyces lilacinus, Th= Seed priming with *Trichoderma hamatum* 5, 10, 15 = duration of bio-priming in minute, 0, 100 & 200 mM NaCl concentration. On bars, vertical lines represent ± Mean Standard Error (S.E) and similar alphabets represents non-significant difference between the means of treatment at *p<0.05*.

Antioxidant enzymes including super oxide dismutase (SOD) and catalase (CAT) activities were measured at different NaCl concentrations with and without endophytes i.e. *P. lilacinus* and *T. hamatum* application. In comparison to the control condition, SOD and CAT activities were stimulated by the degree of salinity stress at 100 mM (44 to 141%) and 200 mM (27 to 110%) in both varieties. However, among the two varieties, the increment of SOD and CAT in wheat was greater in comparison to mung beans. Moreover, among the priming treatments, *T. haamatum* (15 min) prompted the highest SOD (141, 151, 74 and 110%) and CAT (141, 71, 62 and 62%) activity under increasing salt stress over the control, in which the least antioxidant activity was observed. Besides, among different treatments of *P. lilacinus* the highest increment in SOD (44 to 72%) and CAT (40 to 101%) activities was attributed to the 15 min of priming in both varieties. (**Figure 7**).

## Discussion

Due to the changing climate and the increasing assault of abiotic stress, agricultural productivity is heavily curtailed. In the present study two sodium-tolerant biological priming agents, namely *T. hamatum* and *P. lilacinus*, with three priming durations (5, 10, and 15 min) were used. Later seeds were allowed to germinate and grow in a salt-stress environment. It was observed that the root and shoot length of both wheat and mung bean plants declined with the elevating salt stress. It is evident from the literature that salt stress inhibited plant growth in a sub-optimal environment (Dey et al., 2004; Azooz et al., 2013; Fizza et al., 2021; Ansari et al., 2022). The decrease in plant growth is attributed to nutrient imbalance, osmotic, and ionic stress (Iqbal and Ashraf, 2013; Rasool et al., 2013; Alqarawi et al., 2014). In the present study, it was observed that the priming with *T. hamatum* and *P. lilacinus* increased the root and shoot length of both wheat and mung bean plants in a sub-optimal environment (**Figure 2**). The highest and most significant amelioration was observed in mung bean plants by virtue of *Trichoderma* priming. Our findings are in accordance with those of Mastouri et al. (2010) and Rawat et al. (2013), who found that *Trichoderma* isolates mitigate the negative effects of salt stress in several plants. It was reported that *Trichoderma* is symbiotically associated with plants and thus enhances plant growth due to hormonal modulation or molecules closely related to GA_3_ (Iqbal and Ashraf, 2013; Rawat et al., 2013). Thus, *Trichoderma* association also elongates roots, which aids plants in absorbing nutrients and water from the soil and improves their ability to withstand salt stress (Arora et al., 1992). Likewise, some *Paecilomyces* spp. has also enhanced plant growth *via* growth-regulating metabolites like IAA and GA that could work to ameliorate the stress (Bashri and Prasad, 2016; Liu et al., 2019).

Our results, with respect to the decrease in chlorophyll content index (CCI) under salt stress are supported by the findings of Ahmad et al. (2016) for *Cicer arietinum*, and Alqarawi et al. (2014) for *Ephedra alata*. The decrease in pigment content is attributed to several factors, including the detrimental effects of salt stress on chloroplast (Zörb et al., 2009), increased activity of chlorophyllase and the consequent reduction in chlorophyll synthesis (Sultana et al., 1999), and instability of the pigment protein complex (Levitt, 1980). The outcomes also demonstrated the potential of *T. hamatum* and *P. lilacinus* in curtailing the detrimental effects of NaCl on the CCI and induced a significant rise in chlorophyll content in both salt-treated plants and control plants (**Figure 1**). *P. lilacinus* has also been reported to increase the chlorophyll content in carrot plants (Nesha and Siddiqui, 2017). Moreover, *Trichoderma* spp. has also been linked to improvements in the pigment system and the reduction of harmful effects of NaCl, according to Rawat et al. (2011) and Zhang et al. (2013). Compared to control, plants that are primed with *T. hamatum* showed improvement in photosynthetic pigments could be attributed by the synthesis of phytohormones such auxin, gibberellins, and cytokinins (Martínez-Medina et al., 2014; Resende et al., 2014).

Salt stress reduced the stomatal conductance of wheat and mung bean plants which is one of the most common responses of plants to prevent excessive water loss and controls the passage of carbon and water between plants and the atmosphere (Brodribb and McAdam, 2011). However, the priming of *T. hamatum* significantly increased the stomatal conductance over control (un-primed) under extreme salt stress (**Figure 1**). While in wheat plants, stomatal closure was observed to reduce transpiration and conserve water during salt stress. This closure is regulated through the ABA level as well as extensive signal transduction of guard cells induced by *T. hamatum* (Efetova et al., 2007; Joshi-Saha et al., 2011). Therefore, two different behavior of *T. hamatum* priming was observed under high salt stress. In wheat plants, it fosters higher stomatal conductance which could be a strategy to fix more CO_2_ due to a fast growth strategy before the onset of salt stress consequences compared to mung bean plants.

In salt stress, H_2_O_2_ can serve both as a measure of toxicity or that damaged plant cells permanently or it may be a secondary messenger that controls the plant’s antioxidant defense (Gechev et al., 2006). In the current investigation, we discovered that salt stress led to a considerable rise in H_2_O_2_ levels. However, in primed wheat plants, the level of H_2_O_2_ was significantly lower than in mung bean plants. Moreover, the more decrease in H_2_O_2_ level was observed among the plants primed with *T. hamatum*, therefore, we proposed that priming of *T. hamatum* promoted lesser oxidative or cellular damage caused by salt stress which is in accordance with the finding of Güler et al. (2001). Likewise, the other damage marker, MDA content was also lower among the wheat plants over the mung beans, hence, the priming was more effective among the wheat plants. As suggested by earlier studies, salt stress may have an impact on altering the composition of membrane lipids since it caused lipid peroxidation (Samadi et al., 2019). The decrease in MDA content suggested that *T. hamatum* prevented the plant from oxidative damage in comparison to unprimed plants. These findings strongly concur with those of Zhang et al. (2013) who discovered lower levels of lipid peroxidation in cucumber plants under salt stress that had been treated with *T. harzianum*.

Salt stress adversely affects the photosynthetic apparatus of the plants which can be observed through chlorophyll a fluorescence parameter. Chl fluorescence is frequently employed as a measure of photosystem efficiency because it offers important information about the quantum efficiency of photochemistry and heat dissipation (Lichtenthaler and Burkart, 1999). Quantum yield (F_V_/F_M_) and PS II functionality gradually decreased with the increase in exposure time and salt concertation, which negatively affected the membrane stability. This suggests that the PS II reaction center deteriorated under higher stress levels (Lu and Zhang, 2000). However, *T. hamatum* priming significantly enhanced the F_V_/F_M_ and PS II efficiency of stressed plants over control and *P. lilacinus* priming. These outcomes are indicative of *T. hamatum* efficacy to enhance salt tolerance which is linked with the improved PS II functionality in stressed plants. The increase in energy loss (DI_O_/RC) among the control plants exhibited stress damage at the PS II level which was quite higher among the control plants while bio-primed plants had considerably very low dissipation hence, lower damage at PS II level.

According to the findings of Ran et al. (2021), the OJIP curve of the present work showed a decline in I and P values with elevated salt stress. However, the increase in I and P steps in *T. hamatum* and *P. lilacinus* primed plants showed the availability of more active reaction centers (RC) PS II under salt stress in comparison to control (unprimed plants) (Kalaji et al., 2011). This indicated that bio-primed plants were more tolerant to salt stress as their absorbed energy was more efficiently transferred to reaction centers for photochemistry (Tsimilli-Michael and Strasser, 2008; Stirbet and Govindjee, 2011). The decrease in I and P phase under salt stress control (unprimed) plants was due to a bottleneck in electron transfer at the electron acceptor side of the PSI, the increase in cyclic electron flow (CEF) around the PS I is revealed by the decrease in I-P phase (Kono et al., 2014; Hamdani et al., 2015). This has been alleviated *via T. hamatum* priming that mitigate the smooth electron flow between PS II and PS I which resulted in high photosynthetic yield of the stressed and unstressed plants (**Figure 6**).

**Figure 8:**
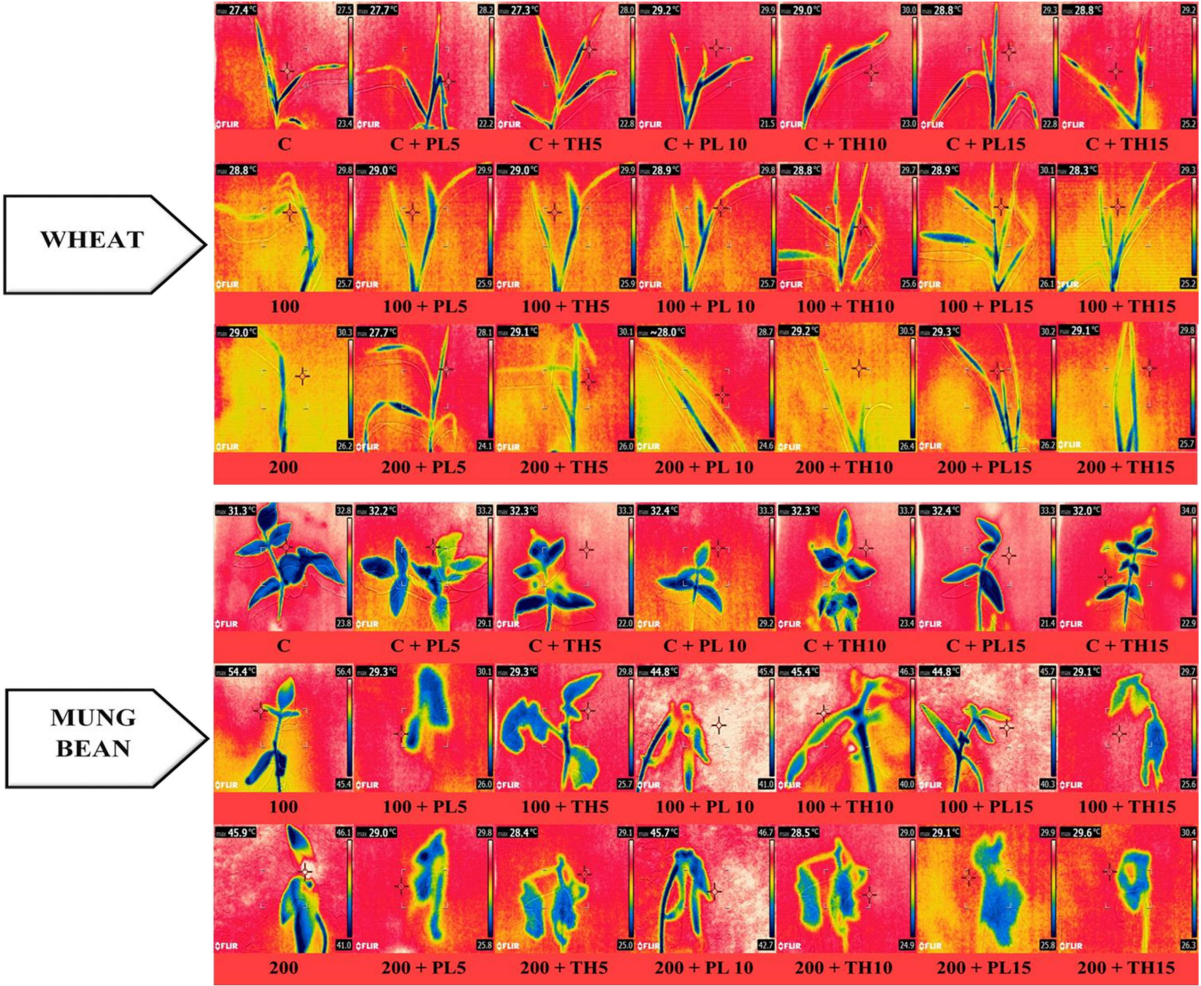
Effects of seed priming with fungal endophytes on infra-red thermal images of wheat and mung bean grown under saline environment. The symbols on the horizontal axis represents: C = Seed without priming, PL5= Seed priming with *Paecilomyces lilacinus* for 5 mins, PL 10= Seed priming with *Paecilomyces lilacinus* for 10 mins, PL 15= Seed priming with *Paecilomyces lilacinus* for 15 mins, TH 5= Seed priming with *Trichoderma hamatum* for 5 mins, TH 10= Seed priming with *Trichoderma hamatum* for 10 mins, TH 15= Seed priming with *Trichoderma hamatum* for 15 mins. 0 (C), 100 and 200 mM represents different salinity (NaCl) levels.

According to the leaf energy flux model (**Figure 8**) the highest absorption per reaction center (ABs/RC) and dissipation per reaction center (DI_O_/RC) were observed among the un-primed plants (wheat and mung bean) which was due to more inactive reaction centers (RC) to active reaction center ratio. Hence, this explains that the controlled plants were able to absorb more photons, but the trapped energy was not used to reduce the plastoquinone pool and absorbed photon was rather dissipated in the form of energy or heat. However, bio-priming enhanced the active to inactive RC ratio among the wheat and mung bean plants which helped to increase the rate of Q_A_ reduction by trapped exciton (TR_O_/RC) under high salt stress (200 mM). This increment led to the enhanced electron transport (ET_O_/RC) which reflected the increased activity of active RC to reoxidize the reduced Q_A_ (Grieco et al., 2015). This combined increased in trapping and transport of exciton displayed the stress tolerance induced by bio-priming agent which reflected in the enhanced photosynthetic yield (PI) and least energy dissipation (DI_O_/RC) of the primed plants.

**Figure 9:**
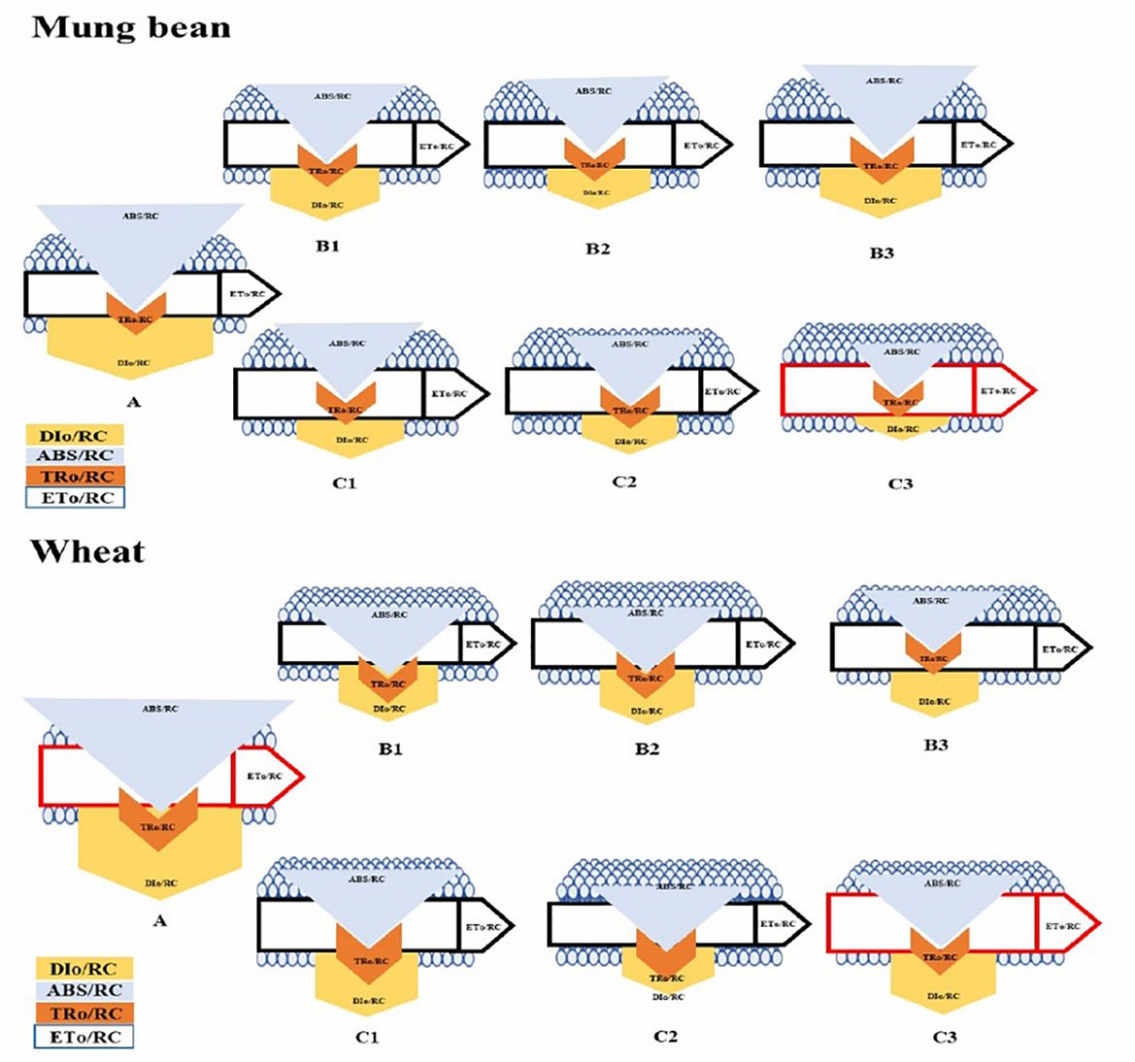
Membrane pipeline model showing the proportion of specific energy fluxes in treated plants. In the membrane, ABS/RC, TRO/RC, ETO/RC, and DIO/RC indicate absorption, maximum trapped exciting flux per active PSII, electron transport, and dissipation flux, respectively. The value of each parameter can be seen in relative changes in the width of each arrow (see the color legend). The diagram exhibits the variation of ABS/RC, TRO/RC, ETO/RC, and DIO/RC, for seven treatments, namely, A=200mM, B1=200mM and *Paecilomyces lilacinus* strain with 5 minutes time interval, B2=200mM and *Paecilomyces lilacinus* strain with 10 minutes time interval, B3=200mM and *Paecilomyces lilacinus* strain with 15 minutes time interval, C1=200mM and *Trichoderma harzianum* strain with 5 minutes time interval, C2=200mM and *Trichoderma harzianum* strain with 10 minutes time interval, and C3=200mM and *Trichoderma harzianum* strain with 15 minutes time interval. The model displays fluxes in different shapes; the size of each shape was developed by the different values of four fluxes in each treatment.

The infra-red thermographic images also evidently supported the results. A significant color change was observed among the leaves of primed and un-primed plants indicating a rise in leaf temperature of the control plants under high salt stress (**Figure 9**). This rise in temerature reflects the decline in water contents of the leaves. It was evident from the data that bio-primed plants demosntrate lesser increase in leave temperature corresponding with higher water content. Moreover, under the water stress, leaf temperature somewhat mimicked the gas exchange rates and grain output, perhaps due to other changes brought on by this stress factor in plants, like impairments in the rates of photosynthesis and partitioning of energy in plant leaves and canopy structures (resulting in variations in the absorption and/or dissipation of energy) (Casari et al., 2019). Therefore, the results were coherent that the bio-primed plants were more tolerant to varying levels of salt stress (0, 100, and 200 mM) in comparison to the control plants.

Antioxidant activities are important physiological aspects playing a key role in coping with salt stress (Guo et al., 2018). Abiotic stress causes an increase in ROS production that must be controlled in a homeostatic pool, yet excessive levels of ROS can produce oxidative stress, which can damage plant physiology and cause plant death by causing denaturation of protein structure, lipid peroxidation, and nucleotide disruption (Demidchik, 2015). In this context, an increase in antioxidant activity protects cells against environmental challenges like salinity and drought. *P. lilacinus &* specifically *T. hamatum* treated plants showed a remarkable increase in antioxidant enzyme activities like SOD and CAT under high salt stress (200 mM). which significantly reduce the production of ROS like H_2_O_2_ that is potent enough to induce lipid peroxidation in cell membrane. Hence, increasing antioxidant activities ultimately brings down the level of MDA in treated plants as compared to control by scavenging ROS (**Figure 7**).

It is concluded that bio-priming with endophytes produces resistant in crop plants to salt stress through modulation in physiological and photosystem II functionality which was further supported by the infrared thermographic images of the stress and control plants. Endophytes not only sustain better quantum absorption and energy flow in plants but also contribute to sustaining photosystem II performance and lower down the stress markers production and energy loss in a sub-optimal environment. Further our current findings suggest that the use of bio-priming with salt tolerant and bio-stimulating natural colonizers specifically with *T. hamatum* could be a suitable approach in mitigating salt stress in wheat and mung bean plants.

## Data availability statement

The original contributions presented in the study are included in the article/**Supplementary Material**. Further inquiries can be directed to the corresponding authors.

## Author contributions

All authors contributed to the study’s conception and design. Material preparation, search, and collection of relevant articles and reviews were performed by KI, ZS, JC, XW, YR, HA and DW thoroughly checked the first draft and decisively improved the manuscript. All authors contributed to the article and agreed the submitted version. All authors contributed to the article and approved the submitted version.
