# Supplementary material for: Bio-priming with salt tolerant endophytes improved crop tolerance to salt stress via modulating photosystem II and antioxidant activities in a sub-optimal environment
Source: Front Plant Sci. 2023 Mar 9;14:1082480. doi: 10.3389/fpls.2023.1082480 (PMC10037113; doi:10.3389/fpls.2023.1082480)
Supplement: Supplementary file 1 [file Presentation_1.pptx]

## Slide 1
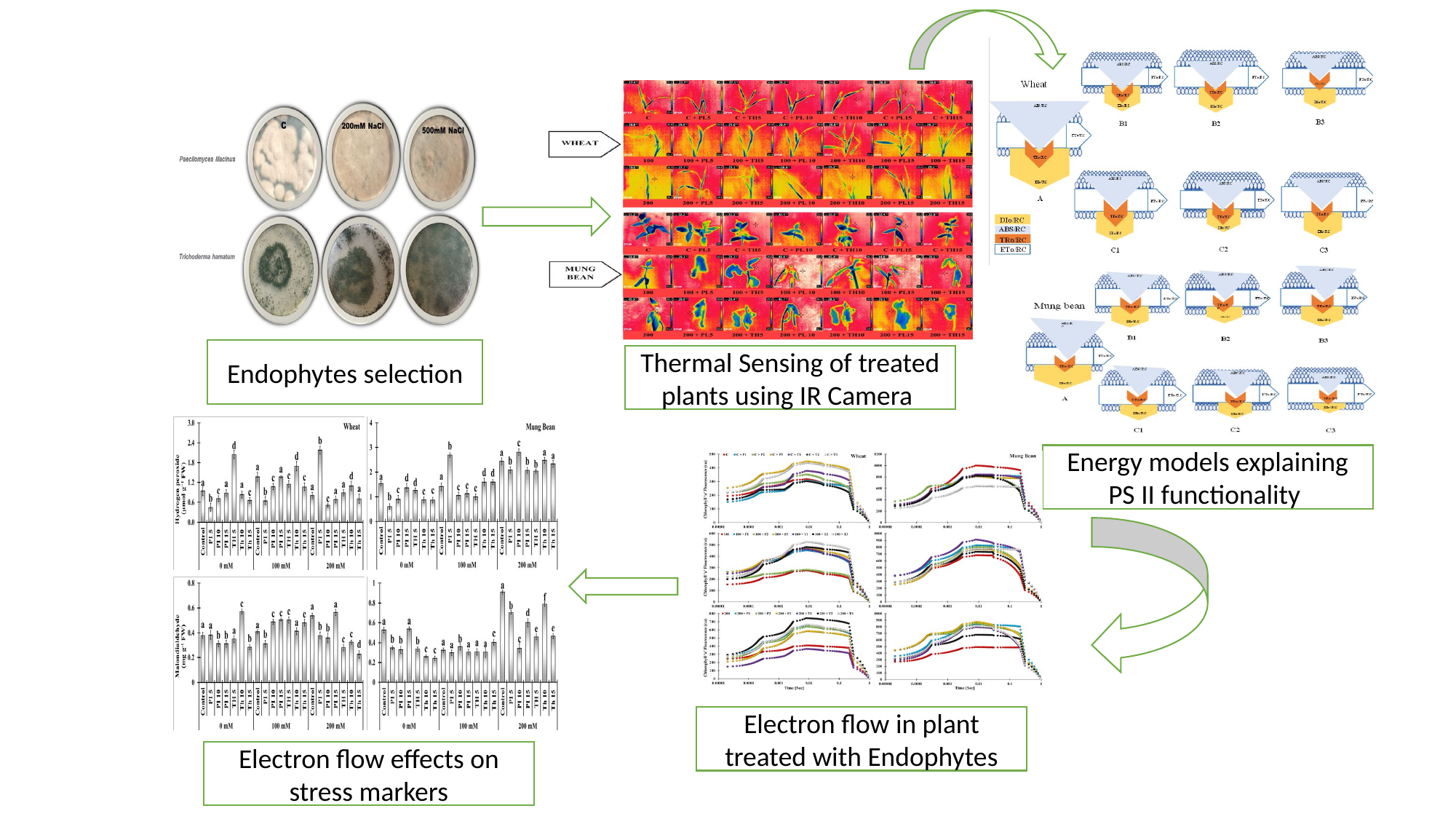

Endophytes selection
Thermal Sensing of treated plants using IR Camera
Energy models explaining PS II functionality
Electron flow in plant treated with Endophytes
Electron flow effects on stress markers
