# Supplementary material for: Bio-priming with salt tolerant endophytes improved crop tolerance to salt stress via modulating photosystem II and antioxidant activities in a sub-optimal environment
Source: Front Plant Sci. 2023 Mar 9;14:1082480. doi: 10.3389/fpls.2023.1082480 (PMC10037113; doi:10.3389/fpls.2023.1082480)
Supplement: Supplementary file 2 [file Table_1.docx]

| **Fluorescence parameters** | **Description** |
| --- | --- |
| F_0_ ≅ F_20μs_ | Minimal fluorescence, when all RCs are open. |
| F_M_ (= F_P_) | Maximal fluorescence, when all RCs are closed (= F_P_ when the actinic light intensity is above 500 μmol photons m^−2^ s^−1^ and provided that all RCs are active as Q_A_ reducing). |
| F_V_ ≡ F_M_ − F_0_ | Maximal variable fluorescence. |
| F_V_ / F_0_ = (F_M_ - F_0_) / F_0_ | The activity of the water splitting complex on the donor site of the PSII. |
| F_V_ / F_M_ = (F_M_ - F_0_) / F_M_ | Maximum quantum yield of PSII. |
| V_J_ = (F_J_ - F_O_) / (F_M_ - F_0_) | Relative variable fluorescence at the J-step. |
| ϕ_Po_ _=_ 1 - F_O_/F_M_ | Maximum quantum yield of primary photochemistry (at t=0). |
| ϕ_Do =_ F_0_/F_M_ | Quantum yield (at t=0) of energy dissipation. |
| PI_ABS_ _=_ ϒ_RC_/ (1 - ϒ_RC_).  ϕ_Po_/ (1 - ϕ_Po_). ψ_Eo_/ (1 - ψ_Eo_) | Performance index (potential) for energy conservation from exciton to the reduction of intersystem electron acceptors. |
| M_0_  ABS/RC = (M_O_/V_J_). (1/φ_Po_) | Approximated initial slope (in ms^-1^) of the fluorescence transient V = f (t).  Apparent antenna size of an active PS II RC. |
| TR_O_/RC = M_0_ (1/V_J_) | Trapping flux (leading to Q_A_ reduction) per RC. |
| ET_O_/RC = M_0_ (1/V_J_)ψ_O_ | Electron transport flux (further than Q_A_) per RC. |
| DI_O_/RC = (ABS/RC - TR_O_/RC) | Dissipated energy flux per RC (at t=0). |

Suplimentary Table 1: Photochemicals tratis and there description which is used in the present study.
