# Supplementary material for: Bio-priming with salt tolerant endophytes improved crop tolerance to salt stress via modulating photosystem II and antioxidant activities in a sub-optimal environment
Source: Front Plant Sci. 2023 Mar 9;14:1082480. doi: 10.3389/fpls.2023.1082480 (PMC10037113; doi:10.3389/fpls.2023.1082480)
Supplement: Supplementary file 3 [file Table_2.docx]

| **TREATMENTS** | **CCI** | **SC** | **RL** | **SL** | **R/S Ratio** | **H_2_O_2_** | **MDA** | **F_V_/F_M_** | **F_V_/F_O_** | **PI** | **F_O_/F_M_** | **F_M_/F_O_** | **Vj** | **M_O_** | **ABS/RC** | **TR_O_/RC** | **ET_O_/RC** | **DI_O_/RC** |
| --- | --- | --- | --- | --- | --- | --- | --- | --- | --- | --- | --- | --- | --- | --- | --- | --- | --- | --- |
|  |  |  |  |  | **Mung-bean** |  |  |  |  |  |  |  |  |  |  |  |  |  |
| **C** | .00 | .00 | .00 | .00 | .00 | .00 | .00 | .00 | .00 | .00 | .00 | .00 | .00 | .00 | .00 | .00 | .00 | .00 |
| **C + P1** | 91.94 | -20.09 | -32.47 | -27.17 | -7.28 | -60.53 | -34.01 | -4.14 | -14.64 | 19.98 | 13.34 | -11.77 | 49.33 | 99.23 | 36.66 | 63.74 | 9.63 | 32.39 |
| **C + P2** | 92.36 | -24.91 | -40.52 | -33.00 | -11.22 | -41.64 | -38.04 | -8.40 | -28.10 | -4.77 | 30.05 | -23.11 | 143.85 | 224.27 | 48.50 | 83.37 | -19.78 | 42.99 |
| **C + P3** | -8.02 | 35.57 | -46.26 | -33.67 | -18.99 | -10.95 | 1.70 | -3.75 | -14.67 | -1.27 | 12.10 | -10.80 | 48.20 | 82.64 | 29.32 | 57.23 | 7.11 | 24.92 |
| **C + T1** | -27.97 | -9.29 | -18.10 | -9.17 | -9.84 | -18.07 | -36.59 | -1.90 | .21 | 47.29 | 5.18 | -4.93 | 69.83 | 115.48 | 27.21 | 51.43 | -5.09 | 23.39 |
| **C + T2** | -10.96 | 18.65 | -6.03 | 7.83 | -12.86 | -43.01 | -50.79 | -1.55 | -5.72 | 20.44 | 4.22 | -4.05 | 58.13 | 115.24 | 37.34 | 64.06 | 10.59 | 33.12 |
| **C + T3** | -3.28 | 34.20 | 2.01 | -8.67 | 11.69 | -43.48 | -54.45 | -15.42 | -25.57 | -4.57 | 31.12 | -23.74 | 37.68 | 86.27 | 85.68 | 66.65 | 22.32 | 88.68 |
|  |  |  |  |  |  |  |  |  |  |  |  |  |  |  |  |  |  |  |
| **100** | .00 | .00 | .00 | .00 | .00 | .00 | .00 | .00 | .00 | .00 | .00 | .00 | .00 | .00 | .00 | .00 | .00 | .00 |
| **100 + P1** | 47.04 | 1.85 | -14.04 | -22.63 | 11.11 | 91.12 | -8.03 | -3.12 | -4.17 | 108.78 | 7.48 | -6.96 | -6.66 | -14.42 | -7.27 | -20.71 | -9.70 | -4.43 |
| **100 + P2** | 132.83 | 6.27 | -17.54 | -26.72 | 12.53 | -25.92 | 10.81 | 4.99 | 19.23 | 77.49 | -11.39 | 12.86 | -9.46 | -12.35 | -7.75 | -12.11 | 3.47 | -6.83 |
| **100 + P3** | 170.56 | -29.64 | .00 | 21.34 | -17.58 | -19.96 | -6.18 | 9.60 | 46.37 | 301.04 | -23.51 | 30.74 | -14.06 | -30.19 | -30.61 | -32.30 | -20.80 | -30.26 |
| **100 + T1** | 36.22 | -7.07 | 33.68 | 34.48 | -.59 | -29.03 | -5.25 | 3.73 | 13.94 | 67.59 | -7.87 | 8.54 | -5.34 | -6.80 | -7.99 | -10.68 | -3.44 | -7.43 |
| **100 + T2** | 74.46 | 30.52 | 19.65 | 14.44 | 4.55 | 13.47 | -6.08 | 3.87 | 14.23 | 10.54 | -8.29 | 9.04 | 11.36 | 3.19 | -10.93 | -7.83 | -15.39 | -11.58 |
| **100 + T3** | 170.56 | 26.51 | 8.42 | 13.79 | -4.72 | 13.58 | 24.82 | 4.38 | 17.25 | 111.56 | -10.04 | 11.16 | -21.54 | -28.18 | -12.70 | -17.79 | 5.00 | -11.62 |
| **200** | .00 | .00 | .00 | .00 | .00 | .00 | .00 | .00 | .00 | .00 | .00 | .00 | .00 | .00 | .00 | .00 | .00 | .00 |
| **200 + P1** | -29.68 | 9.62 | 66.01 | 5.41 | 57.45 | -14.65 | -22.49 | 26.22 | 91.05 | 73.00 | -34.02 | 51.56 | -14.95 | -24.97 | -32.23 | -17.65 | -2.81 | -34.34 |
| **200 + P2** | 35.73 | -8.58 | 204.43 | 18.02 | 157.94 | 14.93 | -62.07 | 20.39 | 57.00 | -5.92 | -26.73 | 36.48 | 33.21 | 11.31 | -34.44 | -18.52 | -36.09 | -36.76 |
| **200 + P3** | 61.81 | 3.52 | 226.60 | 36.30 | 139.63 | -15.13 | -33.71 | 24.85 | 74.51 | -2.59 | -31.52 | 46.02 | 32.56 | 30.86 | -22.12 | 5.20 | -18.61 | -26.09 |
| **200 + T1** | 206.41 | 159.05 | 216.75 | 2.72 | 208.37 | -16.65 | -49.71 | 32.22 | 118.32 | 94.72 | -42.24 | 73.14 | -28.00 | -37.76 | -33.40 | -22.57 | 10.32 | -34.98 |
| **200 + T2** | 134.68 | 65.98 | 197.54 | 23.21 | 141.49 | 1.32 | -13.28 | 22.41 | 66.30 | -8.19 | -28.62 | 40.10 | 12.26 | -12.51 | -38.64 | -19.73 | -27.11 | -41.39 |
| **200 + T3** | 108.17 | -6.10 | 285.71 | 24.69 | 209.34 | -4.56 | -48.72 | 30.08 | 112.89 | 71.83 | -40.00 | 66.66 | -17.70 | -37.34 | -41.54 | -26.74 | -8.59 | -43.69 |

| **TREATMENTS** | **CCI** | **SC** | **RL** | **SL** | **R/S Ratio** | **H_2_O_2_** | **MDA** | **F_V_/F_M_** | **F_V_/F_O_** | **PI** | **F_O_/F_M_** | **F_M_/F_O_** | **Vj** | **M_O_** | **ABS/RC** | **TR_O_/RC** | **ET_O_/RC** | **DI_O_/RC** |
| --- | --- | --- | --- | --- | --- | --- | --- | --- | --- | --- | --- | --- | --- | --- | --- | --- | --- | --- |
|  |  |  |  |  |  |  |  | wheat |  |  |  |  |  |  |  |  |  |  |
| **C** | .00 | .00 | .00 | .00 | .00 | .00 | .00 | .00 | .00 | .00 | .00 | .00 | .00 | .00 | .00 | .00 | .00 | .00 |
| **C + P1** | -44.85 | -46.11 | 44.33 | -11.93 | 63.87 | -52.67 | 1.24 | 14.98 | 44.57 | 193.03 | -16.97 | 20.44 | 9.90 | 35.54 | -1.26 | 14.61 | -14.09 | -3.47 |
| **C + P2** | -22.26 | -55.85 | -7.22 | -49.54 | 83.88 | -24.18 | -17.30 | 1.31 | 14.46 | 4.19 | -5.12 | 5.39 | 14.40 | 121.48 | 89.84 | 65.62 | 22.66 | 93.21 |
| **C + P3** | 31.23 | -42.10 | 130.93 | 42.20 | 62.39 | -6.48 | -17.48 | 9.43 | 34.99 | 160.26 | -13.44 | 15.52 | 28.55 | 67.62 | 15.73 | 16.34 | -23.63 | 15.64 |
| **C + T1** | 9.97 | -18.87 | -25.77 | -31.19 | 7.88 | 117.31 | -7.59 | 33.40 | 110.92 | 132.01 | -35.74 | 55.61 | -6.63 | 50.81 | 11.21 | 52.54 | 39.51 | 5.47 |
| **C + T2** | -40.86 | -50.38 | -12.37 | -29.91 | 25.02 | -11.65 | 51.10 | 2.00 | 20.33 | -51.49 | -15.02 | 17.67 | -62.49 | -50.31 | 19.28 | -9.02 | 42.19 | 45.02 |
| **C + T3** | 199.00 | 5.79 | 54.64 | -3.67 | 60.53 | -30.37 | -24.62 | 27.59 | 83.98 | 271.10 | -29.89 | 42.64 | -23.49 | -3.15 | -3.19 | 11.02 | 29.41 | -5.16 |
| **100** | .00 | .00 | .00 | .00 | .00 | .00 | .00 | .00 | .00 | .00 | .00 | .00 | .00 | .00 | .00 | .00 | .00 | .00 |
| **100 + P1** | -43.84 | -15.62 | -12.40 | 14.23 | -23.32 | -52.96 | -23.84 | 27.62 | 53.58 | 66.30 | -14.68 | 17.20 | 14.12 | -44.26 | 8.24 | -51.42 | 36.15 | .88 |
| **100 + P2** | -42.47 | -17.45 | -18.60 | -2.88 | -16.19 | -21.22 | 19.10 | -6.02 | -25.66 | -40.88 | 28.40 | -22.12 | 26.48 | -37.90 | 49.72 | -46.94 | 36.06 | 44.38 |
| **100 + P3** | -44.29 | 25.46 | -30.23 | -5.77 | -25.96 | -.12 | 23.51 | 16.10 | 44.43 | 86.55 | -11.98 | 13.62 | 53.76 | -36.24 | 6.97 | -56.20 | -6.63 | 1.13 |
| **100 + T1** | 141.10 | -5.12 | 4.96 | 13.08 | -7.18 | -15.91 | 23.02 | 26.83 | 63.14 | 146.64 | -13.79 | 16.00 | 12.14 | -59.56 | -16.88 | -63.68 | 4.08 | -22.20 |
| **100 + T2** | 209.59 | 4.72 | 29.46 | 32.69 | -2.44 | 23.58 | .98 | 41.43 | 115.24 | 394.76 | -34.46 | 52.57 | -.88 | -68.95 | -33.95 | -69.98 | .26 | -38.57 |
| **100 + T3** | 285.84 | 28.48 | 20.16 | 34.62 | -14.20 | -22.07 | 17.63 | 45.22 | 133.79 | 348.40 | -37.08 | 58.94 | -3.90 | -66.53 | -31.16 | -67.59 | 8.19 | -36.37 |
| **200** | .00 | .00 | .00 | .00 | .00 | .00 | .00 | .00 | .00 | .00 | .00 | .00 | .00 | .00 | .00 | .00 | .00 | .00 |
| **200 + P1** | 44.26 | -28.31 | 149.40 | 26.44 | 97.25 | 169.62 | -29.56 | 10.67 | 42.15 | 291.87 | -20.64 | 26.01 | -27.49 | -50.65 | -39.30 | -40.40 | -19.65 | -39.08 |
| **200 + P2** | 83.20 | -17.07 | 20.00 | 18.77 | 1.03 | -36.92 | -32.98 | 14.21 | 56.24 | 267.63 | -26.75 | 36.53 | -13.98 | -42.77 | -42.18 | -41.90 | -26.26 | -42.23 |
| **200 + P3** | 362.70 | -14.65 | 38.55 | 45.59 | -4.84 | -15.63 | 4.98 | 18.26 | 82.31 | 455.16 | -35.26 | 54.45 | -30.40 | -56.26 | -46.59 | -45.52 | -20.87 | -46.80 |
| **200 + T1** | 81.97 | -47.37 | 76.63 | 10.73 | 59.51 | 10.54 | -47.85 | 6.32 | 21.73 | 174.86 | -11.04 | 12.41 | -22.24 | -39.00 | -31.69 | -30.86 | -15.82 | -31.85 |
| **200 + T2** | 189.34 | -32.57 | 55.42 | 28.35 | 21.09 | 35.88 | -39.64 | 15.12 | 62.98 | 247.38 | -28.81 | 40.47 | -6.09 | -36.53 | -42.04 | -38.03 | -30.37 | -42.80 |
| **200 + T3** | 92.62 | -16.50 | 84.34 | 20.69 | 52.74 | -12.20 | -58.00 | 14.72 | 59.45 | 357.97 | -28.40 | 39.67 | -37.97 | -53.81 | -36.16 | -39.76 | -4.48 | -35.47 |
